# Comparative Genomics and Transcriptomics of *Propionibacterium acnes*

**DOI:** 10.1371/journal.pone.0021581

**Published:** 2011-06-27

**Authors:** Elzbieta Brzuszkiewicz, January Weiner, Antje Wollherr, Andrea Thürmer, Jennifer Hüpeden, Hans B. Lomholt, Mogens Kilian, Gerhard Gottschalk, Rolf Daniel, Hans-Joachim Mollenkopf, Thomas F. Meyer, Holger Brüggemann

**Affiliations:** 1 Göttingen Genomics Laboratory, Institute of Microbiology and Genetics, Georg-August-University of Göttingen, Göttingen, Germany; 2 Department of Immunology, Max Planck Institute for Infection Biology, Berlin, Germany; 3 Department of Medical Microbiology and Immunology, Aarhus University, Aarhus, Denmark; 4 Core Facility Microarray, Max Planck Institute for Infection Biology, Berlin, Germany; 5 Department of Molecular Biology, Max Planck Institute for Infection Biology, Berlin, Germany; University of Liverpool, United Kingdom

## Abstract

The anaerobic Gram-positive bacterium *Propionibacterium acnes* is a human skin commensal that is occasionally associated with inflammatory diseases. Recent work has indicated that evolutionary distinct lineages of *P. acnes* play etiologic roles in disease while others are associated with maintenance of skin homeostasis. To shed light on the molecular basis for differential strain properties, we carried out genomic and transcriptomic analysis of distinct *P. acnes* strains. We sequenced the genome of the *P. acnes* strain 266, a type I-1a strain. Comparative genome analysis of strain 266 and four other *P. acnes* strains revealed that overall genome plasticity is relatively low; however, a number of island-like genomic regions, encoding a variety of putative virulence-associated and fitness traits differ between phylotypes, as judged from PCR analysis of a collection of *P. acnes* strains. Comparative transcriptome analysis of strains KPA171202 (type I-2) and 266 during exponential growth revealed inter-strain differences in gene expression of transport systems and metabolic pathways. In addition, transcript levels of genes encoding possible virulence factors such as dermatan-sulphate adhesin, polyunsaturated fatty acid isomerase, iron acquisition protein HtaA and lipase GehA were upregulated in strain 266. We investigated differential gene expression during exponential and stationary growth phases. Genes encoding components of the energy-conserving respiratory chain as well as secreted and virulence-associated factors were transcribed during the exponential phase, while the stationary growth phase was characterized by upregulation of genes involved in stress responses and amino acid metabolism. Our data highlight the genomic basis for strain diversity and identify, for the first time, the actively transcribed part of the genome, underlining the important role growth status plays in the inflammation-inducing activity of *P. acnes*. We argue that the disease-causing potential of different *P. acnes* strains is not only determined by the phylotype-specific genome content but also by variable gene expression.

## Introduction

The Gram-positive bacterium *Propionibacterium acnes* is considered as a skin commensal, which preferentially resides in sebaceous follicles. The bacterium's presence on the human skin is suggested to be beneficial, for instance due to the ability to lower the skin pH by acidic fermentation products, thereby protecting the follicles against colonization by harmful pathogens [1]. However, several lines of evidence indicate that, under certain conditions, *P. acnes* can act as an opportunistic pathogen [1], [2]. The involvement of *P. acnes* in the formation and severity of acne vulgaris is widely accepted, albeit precise mechanistic insight remains scarce. Moreover, *P. acnes* has been detected in various opportunistic infections such as endocarditis and osteomyelitis, and in severe post-surgical infections, e.g. after implantation of prosthetic heart valves and shunts [3], [4]. Initially, the bacterium's presence was considered as contamination or secondary invasion; more recently, however, there is growing awareness that *P. acnes* could be an etiological agent of at least some of these diseases [4]. This assumption has been bolstered by immunological observations *in vitro*, demonstrating that *P. acnes* possesses extensive immunostimulatory activity that triggers the secretion of proinflammatory cytokines and chemokines, the activation of the complement system and the stimulation of T-cells [5], [6], [7], [8]. *P. acnes* strains belonging to different phylotypes are reported to differ in their immunostimulatory activity; for instance, strains vary in their ability to trigger human-beta-defensin 2 in keratinocytes, and in their effect on differentiation and viability of sebocytes [9], [10], [11], [12]. To date, bacterial traits and factors responsible for triggering and modulating host cell responses have not been uncovered.

*P. acnes* strains were categorized as phylotypes IA, IB, II and III according to sequence comparison of their *tly* and *recA* genes [13]. More recently, a multilocus sequence typing (MLST) approach, based on nine housekeeping genes [14], has been used to further discriminate strains, resulting in the identification of 57 sequence types (ST) from 210 strains analyzed. Again, three divisions were identified (I, II and III); division I was further subdivided into I-1a, I-1b and I-2. Subdivision I-1a comprised significantly more isolates associated with moderate to severe acne, while strains from other (sub)divisions were isolated more often from healthy skin or opportunistic soft tissue infections. Subdivision I-1a also included the epidemic clone ST18 and its descendents; interestingly, 60% of all ST18 strains have been isolated from acne patients, suggesting that ST18 strains possess a particular virulence potential [14].

Due to the relative scarcity of available *P. acnes* genome sequences, the genetic basis for the heterogeneity of *P. acnes* has not yet been studied on the genomic level. Here we sequenced strain 266, a type I-1a strain belonging to ST18, and used its genome sequence for comparative genomic analyses. We show that the main differences between genomes of different *P. acnes* phylotypes are located within four large genomic regions with island-like characteristics, as well as a few smaller genomic regions. Moreover, we noted subtle differences generated by point mutations and potentially also by phase variation, affecting in particular the expression of adhesins. We also carried out comparative transcriptomic analysis of two *P. acnes* strains and monitored growth phase-dependent transcription. Our data provides insight in the metabolic pathways utilized by *P. acnes* and suggests that different strains of *P. acnes* employ distinct energy-conserving strategies; moreover, strain and growth phase differences in the expression of virulence-associated traits were uncovered.

## Results and Discussion

### Phylotyping of sequenced *P. acnes* and general features of the genomes

Currently (January 2011), three *P. acnes* genomes have been completely sequenced (Table 1): strain KPA171202 (KPA) [15], strain SK137 and the human isolate 266. The latter strain was isolated from a pleuropulmonary infection. According to the recently established MLST scheme [14], strain KPA belongs to division I-2 (ST34), whereas SK137 and 266 are both I-1a strains, albeit being different STs (Table 1). Strain 266 is an ST18 strain, which represents an evolutionary lineage comprising isolates frequently associated with mild to severe acne. Additional *P. acnes* genomes are in the gap closure/finishing phase, including the strains J139 (II/new ST), J165 (I-1a/ST18) and SK187 (I-1a/new ST), which alongside strain SK137 (I-1a/new ST) and 67 other *P. acnes* strains (draft assembly) serve as reference genomes for the Human Microbiome Project [16]. The *P. acnes* genomes have a similar GC content (60%) and size, on average 2,509 kb, with little variation (standard deviation: 30.2 kb). The genome of *P. freudenreichii* is 105 kb larger than the average *P. acnes* genome and has an elevated GC content (67%); however, the number of coding sequences (CDS) is similar to *P. acnes* [17].

**Table 1:** Genome features of propionibacteria.

Strain	Phylotype a	Length	GC Content	CDS	DNA coding sequence	GenBank
***P. acnes*** ** 266**	**I-1a/ST18 (IA)**	2,494,578	60%	2,412	90%	this work
***P. acnes*** ** KPA171202**	**I-2/ST34 (IB)**	2,560,265	60%	2,297	89%	AE017283.1 [15]
***P. acnes S*** **K137**	**I-1a/new ST (IA)**	2,495,334	60%	2,352	88%	CP001977.1
***P. acnes*** ** SK187** b	**I-1a/new ST (IB)**	2,510,934	59%	2,381	88%	ADJM00000000
***P. acnes*** ** J165** b	**I-1a/ST18 (IA)**	2,500,083	60%	2,403	88%	ADJL00000000
***P. acnes*** ** J139** b	**II/new ST (II)**	2,481,963	60%	2,364	88%	ADFS00000000
***P. freuden-reichii*** ** ssp. ** ***Shermanii*** ** CIRM-BIA1**	**-**	2,616,384	67%	2,375	86%	FN806773.1 [17]

a multilocus sequence analysis according to [14]. In brackets: typing according to previous method [13]. It is noteworthy that the subgroup IB does not constitute a homogeneous group; thus, strain SK187 is now allocated to subdivision I-1a.

b these genomes are currently not closed: J165, 62 contigs; SK187, 37 contigs; J139, 7 contigs (January 2011).

### Comparative genomics: strain-specific gene content and genomic regions with island-like features

**Figure 1:**
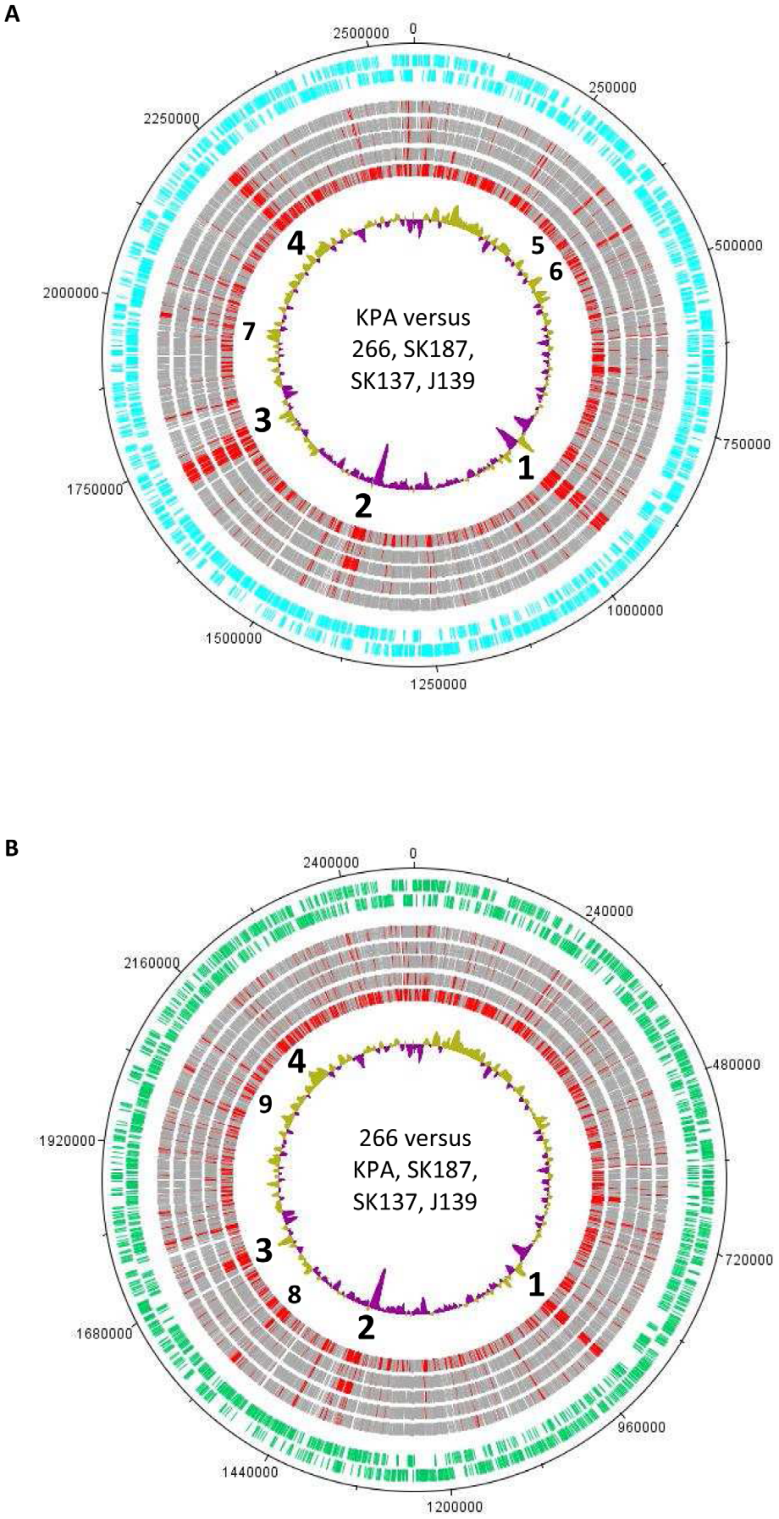
Comparison of five *P. acnes* genomes. (A) All CDS of the reference genome of strain KPA171202 are shown in the two outer rings (cyan, depending on strand location). The subsequent rings depict Bi-Blast results for each CDS of the reference genome in the following genomes (exterior to interior): 266, SK187, SK137, J139 and *P. freudenreichii* (the unfinished genome of *P. acnes* J165 was not taken into consideration due to insufficient quality of CDS prediction). The innermost ring depicts the GC content variation from the mean (60%) of the reference genome. CDS of the KPA genome which have a Bi-Blast hit with > = 25% protein sequence identity in the subject genome are depicted in grey, those with a protein sequence identity <25% are shown in red. (B) The genome of strain 266 was taken as the reference genome (two outer rings in green). The subsequent rings, depicting Bi-Blast hits of each CDS of the reference genome are (exterior to interior): KPA, SK187, SK137, J139 and *P. freudenreichii.* The numbers 1 to 9 represent main genomic islands identified in the reference genomes (see Table S1).

Bi-Blast and direct genome comparisons identified strain-specific regions in the genomes of KPA, 266, SK137, SK187 and J139. All sequenced *P. acnes* isolates share a large core genome (Figure 1). According to a Bi-Blast analysis, 7–11% of the CDS in the five genomes are strain-specific. Comparative analysis identified four large genomic regions within the KPA genome, which are absent from some or all of the other *P. acnes* genomes (Figure 1A). These regions were predicted as genomic islands, possibly acquired by horizontal transfer. In general, most, but not all, predicted islands were specific to a certain *P. acnes* strain or phylotype (Figure S1, Table S1).

The first region in the KPA genome comprises the genes PPA0846-PPA0876, which encode many hypothetical proteins and proteins with similarities to those involved in bacteriocin/lanthionine biosynthesis. This region is also present in SK187 (I-1a), but absent from the other *P. acnes* genomes. The region is flanked by mobile genetic elements: the 5′ end is composed of a *tra*-like region reminiscent of conjugative plasmids, and the 3′ end by a transposase-encoding gene. This underlines its possible horizontal acquisition as predicted by bioinformatics analysis (Figure S1). Several CDS (PPA0859-PPA0865) within this island were identified in a bioinformatics search for genes involved in the biosynthesis of thiazolylpeptides [18]. Thiopeptide antibiotics are potent inhibitors of protein synthesis in Gram-positive bacteria. They are synthesized from a precursor peptide that is subsequently transformed by posttranslational modifications into the final macrocyclic structure. Two precursor peptides are encoded in the *P. acnes* genome, located up- and downstream of the predicted PPA0866 gene; one precursor possesses the 15-aa structural peptide of berninamycin from *Streptomyces bernensis* [18]. The upstream CDS exhibit similarities to the biosynthesis pathways of nosiheptide of *Streptomyces actuosus* (encoded by *nos* genes) [19], siomycin A of *Streptomyces sioyaensis* (*sio* genes), nocathiacin of *Nocardia* sp. (*noc* genes) and in particular TP-1161 of *Nocardiopsis* sp. TFS65-07 (*tpa* genes) [20]; e.g. PPA0859 to TpaK/NocE/NosE/SioJ (24–35% protein identity); PPA0860 to TpaL/NocD/NosD/SioK (31–33% identity) and PPA0862 to TpaC/NocG/NosG (29–47% identity).

The second region (PPA1278-PPA1304) in the KPA genome encodes a gene cluster (PPA1284-PPA1292) with homology to non-ribosomal peptide synthetases (NRPS) and genes involved in the synthesis of cyclic lipopeptides such as surfactin. This cluster is absent in SK137 (I-1a) but present in the other I-1a strains (266, SK187). Thus, this cluster could serve as a marker to further subdivide I-1a strains. There is also an indication that this region was acquired by horizontal transfer: the 5′ end contains PPA1280, which encodes a ParA domain protein with similarities to known plasmid partitioning proteins, indicating that this genomic region might be acquired via plasmid insertion (Figure S1).

The third genomic region (PPA1579-PPA1613), specific to KPA, encodes proteins with homologies to phage proteins, thus is likely to be a cryptic phage. The phage-related genes exhibit no homology to the sequenced *P. acnes* bacteriophage PA6 [21]. It is inserted into a gene encoding a type II restriction enzyme (PAZ_c16690 in strain 266); the remaining gene fragments flank the island (PPA1578, PPA1614).

Several genes of the fourth region (PPA2055-PPA2092) are found only in strain KPA. The genes encode two transport systems, one possibly specific for dipeptides and the other for cobalt, and genes encoding poly-/oligosaccharide-degrading enzymes such as a putative β-glucanase (PPA2056), a putative α-L-fucosidase (PPA2070), and a putative α-galactosidase (PPA2057).

A PCR analysis was carried out to investigate how these four large genomic regions are distributed in a variety of strains (Table 2). It became apparent that the presence and absence of these regions are consistent in strains belonging to the same phylotype: All tested I-1a/ST18 strains possessed only island-like region 2, whereas other STs of the subdivision I-a possessed additional regions also; however, only I-2 strains contained all four genomic regions.

**Table 2:** PCR-detection of island-like genomic regions in *P. acnes* isolates.

	1 (a)	1 (b)	2 (a)	2 (b)	3 (a)	3 (b)	4 (a)	4 (b)	
strain									phylotype
266			+	+					I-1a/ST18
SK137									I-1a/new ST
SK187	+	+	+	+					I-1a/new ST
KPA171202	+	+	+	+	+	+	+	+	I-2/ST34
J139			+	+	+	+			II/new ST
1.4.L1			+	+					I-1a/ST18
12.1.L1			+	+					I-1a/ST18
32.1.L1			+	+					I-1a/ST18
4.1.A1			+	+					I-1a/ST20
12.1.R1			+	+					I-1a/ST20
19.1.R1	+	+	+	+			+	+	I-1a/ST21
3.5.R1			+	+	+	+			I-1a/ST27
2.3.A1	+	+	+	+	+	+	+	+	I-2/ST35
21.1.L1	+	+	+	+	+	+	+	+	I-2/ST36
3.3.R1	+	+	+	+	+	+	+	+	I-2/ST36
10.1.R1			+	+					II/ST52
34.1.A1			+	+					II/ST54
CCUG35547		+		+			+	+	III/ST44
CCUG35900		+		+			+	+	III/ST43
P6	+	+	+	+	+	+	+	+	I-2/ST33

Two PCRs (a and b) per island were performed to check for the presence of the four main genomic islands in the listed human isolates (islands in SK137, SK187 and J139 were predicted from their genomes).

In contrast to KPA, the genomes of the I-1a strains 266, SK137 and SK187 possess only a few strain-specific genomic clusters (Figure 1B). Although the large island-like regions of strain KPA are absent in the genome of strain 266 (with the exception of the NRPS gene cluster, region 2), a signature of genome plasticity (aberrant GC content), can still be detected in the vicinity of these genomic regions, indicating that these regions are hotspots of genomic flexibility/rearrangements. Some regions are restricted to type I-1a genomes; for example, PAZ_c08910-PAZ_c09070 is common to strains 266 and SK137 but is absent in the genomes of the other three strains (Table S1). This region replaces region 1 of strain KPA; besides hypothetical proteins it encodes an efflux ABC transport system and a two-component system. Another region (PAZ_c15380-PAZ_c15460, region 8 in Figure 1B) is specific to the three type I-1a strains and encodes glycosyl hydrolases such as a chitinase-like enzyme (PAZ_c15460) and an ABC transport system belonging to the subfamily specific for the transport of dipeptides, oligopeptides and nickel. Apart from these few differences, there are no additional strain-specific genes in the genome of strain 266 with a reported or predicted function in virulence.

Interestingly, strain SK137 possesses two strain-specific and island-like gene clusters not found in any other sequenced *P. acnes* (depicted as A and B in Figure S1 and Table S1). The first cluster encodes diverse functions such as a recombinase, an ABC transport system, and an N-acetylmuramoyl-L-alanine amidase (autolysin). The second genomic region (20.8 kb) includes a gene cluster encoding proteins related to streptolysin biosynthesis (HMPREF0675_3181-3188); CDS with significant homology (26–31% protein sequence identity) to SagBCD of streptococcal species can be found here (HMPREF0675_3183-85) [22], as well as an efflux ABC transport system with similarity (34% and 35% protein sequence identity, respectively) to MtrA (HMPREF0675_3187) and MtrB (HMPREF0675_3188). This system is essential for resistance to the antitumor agent mithramycin in its producer *Streptomyces argillaceus* [23]. Thus, it is likely that strain SK137 is able to produce a toxin similar to streptolysin and protects itself by employing an efficient export system.

Smaller strain-specific regions exist in all genomes (Table S1); for example, the KPA genome harbors the gene cluster PPA0372-PPA0382 (region 6 in Figure 1A), which differs considerably between KPA and I-1a strains. This region encodes among others a hyalorunate lyase (PPA0380), which is an enzyme predicted to cleave hyaluronan, a non-sulfated glycosaminoglycan and a major component of the extracellular matrix of connective tissues.

In addition to the presence and absence of strain-specific genes, the genomes were found to harbor genes carrying potential frameshift mutations. The orthologs of 34 genes from strain KPA are frameshifted in strain 266, whereas the genome of strain KPA possesses only 14 frameshifted open reading frames (ORFs), whose orthologs in strain 266 are intact (data not shown). This indicates a slightly accelerated gene loss in strain 266, which might be due to reductive genome evolution. Among the frameshifted genes several putative virulence determinants were found, including two mutated genes in strain 266 encoding putative lysophospholipases (PPA0594, PPA1425). By contrast, a lipase (PAZ_c18730) of strain 266 is frameshifted in KPA; this lipase is 45% identical to the characterized glycerol-ester hydrolase A (GehA, PPA2105, PAZ_c21800) [24]. Substantial differences also exist in genes encoding putative adhesins such as proteins with thrombospondin type 3 repeats (Table S1).

### Whole genome gene expression analysis of *P. acnes*

Genomic differences constitute one possible reason for differential strain properties; however, this gives only a superficial picture as regulatory events occurring on the transcriptional and translational level are ignored. Therefore, we also determined the transcriptome of *P. acnes,* focusing on two sets of experiments: (i) determining differential expression in two phylogenetically distinct strains, and (ii) recording growth phase-dependent gene expression changes.

Microarray technology was used for these experiments, utilizing whole genome microarrays that comprised 39,094 oligomers that were derived from the genome of strain KPA. All data can be accessed at http://bioinfo.mpiib-berlin.mpg.de/pacnes/.

### Transcriptional differences between two phylogenetically distinct strains of *P. acnes*

**Figure 2:**
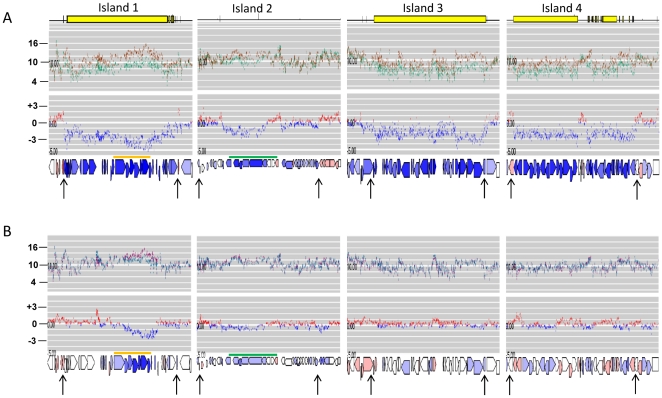
Transcription of the four large island-like genomic regions of *P. acnes.* The transcriptional profiles of the main four genomic islands are shown. The upper panels (A) represents the expression profiles in strain KPA compared to strain 266. The yellow bar depicts the genomic regions that are absent in strain 266 (islands 1, 3 and 4). The first profile depicts the log_2_ intensities (brown, KPA; green, 266), and the second profile the log_2_ ratios (blue, higher in KPA; red, higher in 266). Note that the predicted non-ribosomal peptide biosynthesis gene cluster (PPA1284-1292, green bar) of island 2 is upregulated in strain KPA, albeit present also in strain 266. The lower panel (B) depicts the growth phase-dependent transcription of the genomic regions in strain KPA. Again, the first profile depicts the log_2_ intensities (lilac, exponential phase; cyan, stationary phase), and the second profile the log_2_ ratios (blue, higher in exponential phase; red, higher in stationary phase). Note that the predicted thiopeptide synthesis gene cluster (PPA0859-PPA0866, orange bar) of island 1 is strongly transcribed and upregulated in the exponential growth phase of KPA. The black arrows mark the boundaries of each island-like genomic region.

To obtain additional insight into differing lifestyle and/or virulence properties of *P. acnes* strains, we determined the extent of transcriptional variation between two strains. The transcriptomes of strains KPA (I-2, ST34) and 266 (I-1a, ST18), grown under identical conditions (brain heart infusion (BHI) broth, anaerobic conditions) to mid and late exponential growth phases (OD_600_ 0.3 and 0.6, respectively) were compared. About 100 genes of the strain-specific gene content of strain KPA (located mostly in the island-like genomic regions 1, 3 and 4) were transcribed in the exponential growth phase, and were, therefore, part of the ‘active’ genome (Figure 2). In subsequent analyses strain-specific genes of the two genomes were excluded so that gene expression differences in the common gene pool alone could be determined. This was achieved by considering the data from oligomers of the microarray that matched 100% to both genomes (75.7% (n = 29,587) of all oligomers). The expression of 119 common genes differed (fold-change cutoff 2) between the two strains grown to mid-exponential growth phase, i.e. 37 and 82 genes upregulated in KPA and 266, respectively (Table S2A). In the late exponential growth phase, 316 genes, present in both genomes, were deregulated (Table S2B). It was evident that approximately 27% of these differentially transcribed genes were also deregulated between the mid-exponential and stationary growth phase in strain KPA, thus were expressed in a growth phase-dependent manner (see section “Growth phase-dependent transcription”). This could indicate that the two strains differ in their growth properties. Indeed, the recorded growth curves showed that strain 266 had a slightly shorter doubling time and reached the stationary phase earlier than strain KPA, when grown in BHI medium (data not shown).

### Growth phase-independent transcriptional differences between strains KPA and 266 affect metabolic pathways and secreted factors

**Figure 3:**
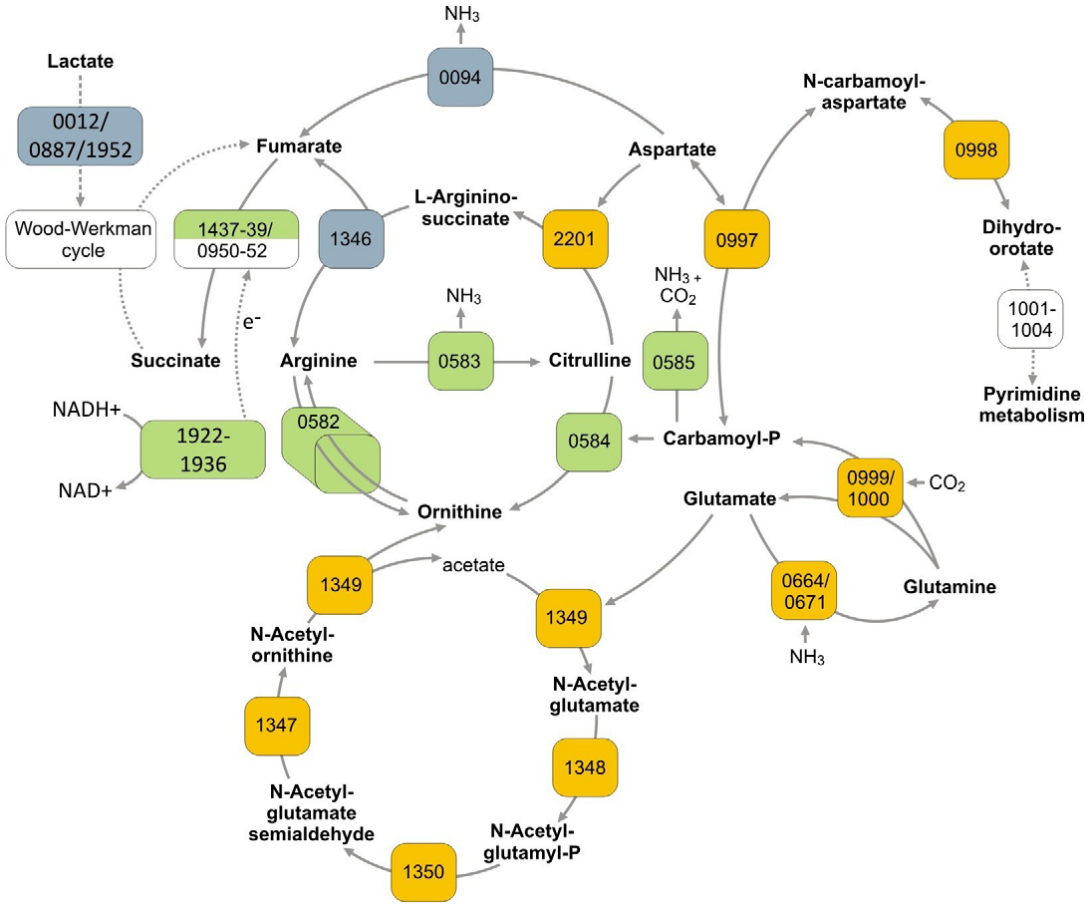
Growth phase-dependent utilization of amino acid-metabolizing pathways in *P. acnes.* The schematic representation includes amino acid-metabolizing enzymes whose genes were strongly deregulated between the exponential (green) and stationary (yellow) growth phases. The arginine deiminase pathway (PPA0582-0585) is an exponential phase trait, whereas the pathways for the interconversion of glutamate to ornithine (PPA1347-1350) and of glutamine/aspartate to dihydroorotate (PPA0997-1000) are stationary growth phase traits. Please note also the connected fumarate-producing reactions selectively upregulated in strain 266 (in grey). Fumarate respiration is a major source of energy conservation in *P. acnes*; most likely, reducing equivalents are delivered by the NADH dehydrogenase (PPA1922-1936). The enzyme-attributed numbers correspond to the gene nomenclature of the KPA genome. For simplicity reasons inorganic and organic phosphate cosubstrates were omitted from the scheme.

Various biological processes associated with differentially expressed genes between strains KPA and 266 were identified using KEGG (Table S3A). Substantial expression differences involved genes implicated in energy metabolism and protein biosynthesis; these were also transcribed in a growth phase-dependent manner (see below). When growth phase-independent expression differences between the two strains were considered, genes encoding transport systems were an overrepresented class: at least five ABC transport systems, two phosphotransferase systems and three other transporters were differentially transcribed in the two strains. Another class of deregulated genes encoded those with various metabolic functions; strain 266 apparently possesses an extended anaerobic metabolic versatility as compared to KPA, in particular employing fermentative pathways to utilize amino acids. For instance, genes encoding pathway components to degrade histidine to glutamate (PPA2166/PPA2167), to process threonine to glycine (PPA0402/403) and to interconvert aspartate, homoserine and lysine (PPA0642, PPA1258, PPA1470, PPA1998) were all upregulated in strain 266. In addition, transcription of genes encoding enzymes that provide the electron acceptor for anaerobic respiration, fumarate, was affected: argininosuccinate lyase (PPA1346) and aspartate-ammonia lyase (PPA0094) were both 3.5-fold upregulated in 266 (Table S2B, Figure 3). Another peculiarity of strain 266 was the increased expression of two lactate dehydrogenases (PPA0012, PPA0887), whose genes are not clustered. Thus, lactate seemed to be a major carbon source of strain 266, possibly taken up via L-lactate permease (PPA0166). The latter gene is clustered with genes of unknown functions (PPA0166-0169), which are 6- to 7-fold upregulated in strain 266; the corresponding proteins are predicted to be iron-sulfur cluster containing proteins, and therefore may be involved in electron transport processes. The gene cluster, PPA0463-0465, which encodes proteins related to *myo*-inositol utilization, was also strongly induced, indicating that *P. acnes* 266 can use *myo*-inositol as a carbon and energy source.

Regarding possible virulence factors, the *gehA* gene (PPA2105) encoding the secreted triacylglycerol lipase [24] is among the up-regulated genes in strain 266. In addition, the genes for the secreted factors endoglycoceramidase (PPA0644) and the adhesin PA-25957 (PPA2127) are 4- and 6-fold upregulated in strain 266, respectively. The latter has been characterized as a host cell-surface attachment protein with dermatan sulfate-binding activity [25]. By contrast, the gene for the Christie-Atkins-Munch-Peterson factor 4 (*camp4*, PPA1231), and the secreted lysozyme M1 (PPA1662) are 8- and 12-fold upregulated in strain KPA, respectively. This is in striking agreement with the findings of our previous proteomic study, which showed that strain 266 exhibited (i) reduced lysozyme M1 secretion, (ii) increased secretion of the lipase GehA and (iii) exclusive secretion of PPA2127, whereas CAMP4 factor was secreted only by KPA [26]. In addition, expression of *htaA* (PPA0786, PA-4687), encoding an iron acquisition protein, was 5-fold increased in strain 266 as compared to KPA; HtaA has been shown to be a major immunoreactive protein of *P. acnes* [25]. The gene encoding the well-characterized polyunsaturated fatty acid isomerase PAI (PPA1039), which catalyzes the isomerization of linoleic acid to 10,12-conjugated linoleic acid (CLA) was also 6-fold upregulated in 266. CLAs are reported to have beneficial effects for the human host due to their apparent antioxidant and anti-cancer properties [27].

Taken together, the transcriptomes of the two investigated strains showed marked differences, in particular with regard to metabolic pathways but also in putative host-interacting properties. These data could provide a molecular basis for the observed differences of *P. acnes* phylotypes in terms of their inflammatory potential [10], [11], [12], [28]. It also supports the previous assumption that certain *P. acnes* strains, i.e. type I-1a strains, are more prone to cause skin disorders than others, i.e. type I-2 strains. However, future work is required to elucidate the factors responsible for this differential behavior, and the underlying mechanisms involved.

### Growth phase-dependent transcription

Virulence properties of most pathogenic bacteria differ between their growth phases. It has been speculated that stress responses, often boosted in the onset of the stationary phase, might be a contributing factor to the immunostimulatory properties of *P. acnes* [29]. Thus, we recorded growth phase-dependent transcription by determining the gene expression differences between an exponential (OD_600_ 0.3) and a stationary (OD_600_ 0.8) growth phase culture of strain KPA, grown under anaerobic conditions in BHI medium. 25% (601 genes) of all genes of the genome were at least 1.5-fold deregulated between the two growth phases. Table S4 lists all genes deregulated at least 2-fold: 137 genes upregulated in the exponential growth phase (EP genes), and 122 genes upregulated in the stationary phase (SP genes). 57% of the EP genes could be mapped to KEGG pathways, whereas only 31% of the SP genes were mapped, indicating an elevated presence of unknown functions in the stationary phase (Table S3B). The most highly enriched pathways among the EP genes were ‘ribosome’ (24 genes), ‘oxidative phosphorylation’ (17 genes), ‘pyrimidine and purine metabolism’ (12 genes), ‘protein export/secretion’ (6 genes) and ‘glycolysis/gluconeogensis’ (5 genes); this reflects, as expected, highly active metabolism, replication machinery and protein translation during the EP. By contrast, the SP was characterized by the upregulation of genes involved in amino acid metabolism (alanine, arginine, aspartate, glutamate and proline metabolism; 12 genes) and diverse transport functions (ABC transporters; 8 genes).

### Growth phase-dependent use of energy-conserving routes

Although *P. acnes* is regarded as an anaerobe, it can also grow under low oxygen tensions. The methylmalonyl-CoA pathway (Wood-Werkman cycle) of propionate formation employed by *P. acnes* and other Propionibacteria grown under anaerobic conditions has been well studied; the main energy-conserving step is accomplished by electron transport to fumarate, a process termed fumarate respiration, and catalyzed by the fumarate reductase (SdhABC) [30]. To date, however, knowledge regarding other energy-conserving strategies of *P. acnes* in response to changing conditions and stress, in particular varying oxygen concentrations, remains fragmentary.

Here, the transcriptome revealed that despite bacterial growth under anaerobic conditions, expression of the whole respiratory chain could be detected. Strong up-regulation of the NADH dehydrogenase/complex I (PPA1922-1936) was detected in the EP, indicating a necessity to re-oxidize NADH, which accumulates during glycolysis and the catabolism of other substrates. Upregulation of genes encoding the cytochrome *bd* oxidase (PPA0175, *cydB*, PPA0176, *cydA*), a respiratory endoxidase, which, in *Escherichia coli*, favors survival under low oxygen tensions [31], was detected in the EP. By contrast, the cytochrome *c* oxidase (PPA0701, *coxB*, PPA0702, *coxA*, PPA1561, *cyoE*) was slightly strongly expressed in the SP. Interestingly, cytochrome *c* reductase (PPA0710-0712) and oxidase are absent from the genome of *P. freudenreichii* [17], indicating that *P. acnes* has a greater ability to react to changing oxygen conditions. We also noted the differential expression of alternative forms of the succinate dehydrogenase/fumarate reductase (SdhABC): the gene cluster PPA1437-39 (*sdhABC*) was strongly upregulated in the EP, whereas PPA0950-52 (*sdhA’B’C’*) was slightly strongly expressed in the SP.

These data indicate that *P. acnes* employs oxidative phosphorylation to conserve energy, employing alternative electron transport routes, and possibly utilizing different electron acceptors. A likely scenario is that reducing equivalents (in the form of NADH) are fed via complex I into the respiratory chain in the EP. Electrons are transferred via cytochrome *b* to SdhABC, which acts as a fumarate reductase, and uses fumarate as a terminal electron acceptor. Thus, proton translocation could be achieved by two membrane-bound systems (complex I and SdhABC); ATP is generated via a F_O_F_1_ ATP synthase (PPA1238-1245), which is also partially upregulated in the EP. It seems plausible to assume that the Wood-Werkman cycle and the respiratory chain are interconnected via fumarate reductase. The key enzyme of the cycle, methylmalonyl-CoA carboxytransferase (PPA2007/2008), is highly expressed in both growth phases. Due to the use of the Gas-Pak anaerobic system for culturing *P. acnes* in this study, we cannot exclude the possibility that residual oxygen was present, which could be used by the cytochrome *bd* oxidase. However, expression of genes of the respiratory chain did not differ between *P. acnes* grown in the Gas-Pak system from bacteria grown under a defined and constant anaerobic atmosphere (data not shown). The employment of terminal electron acceptors other than oxygen, such as nitrate, is very likely; indeed, a nitrate reductase (PPA0507-PPA509) was strongly upregulated in the EP. It remains unclear why the expression of a different fumarate reductase (SdhA'B'C') and a cytochrome *c* oxidase is upregulated in the SP. One possible explanation is that this branch of the aerobic respiratory chain is part of a stress response in the SP that prepares the bacteria to react to changing environmental conditions, i.e. increasing oxygen concentrations.

### Metabolic peculiarities: deregulation of arginine metabolism

**Figure 4:**
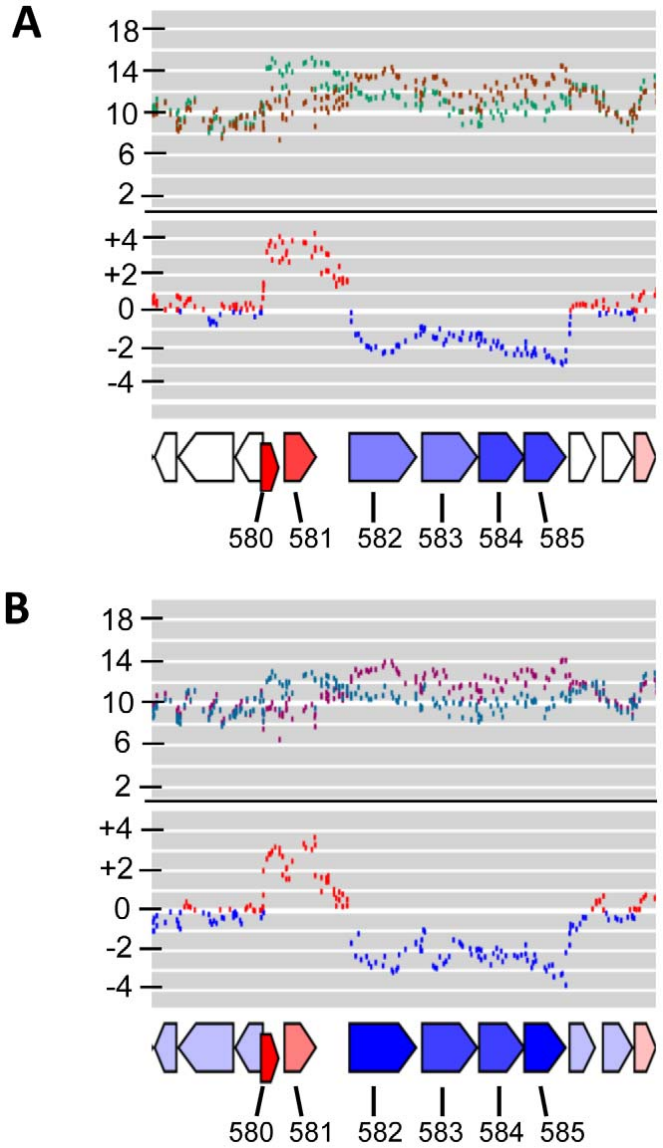
The expression profile of the arginine deiminase gene cluster of *P. acnes.* The most highly deregulated genes in the entire *P. acnes* transcriptome are shown here. The gene cluster PPA0582-0585 encodes the arginine deiminase pathway, and PPA0580/PPA0581 encode stress response genes. Note the opposing expression of PPA0580/0581 and PPA0582-0585. The upper panels (A) represents the expression profiles in strain KPA compared to strain 266. The first profile depicts the log_2_ intensities (brown, KPA; green, 266), and the second profile the log_2_ ratios (blue, higher in KPA; red, higher in 266). The lower panel (B) depicts the growth-phase dependent transcription of this genomic region in strain KPA. The first profile depicts the log_2_ intensities (lilac, exponential phase; cyan, stationary phase), and the second profile the log_2_ ratios (blue, higher in exponential phase; red, higher in stationary phase).

Among the biological processes that were most strongly deregulated between the two growth phases, pathways of amino acid metabolism were prominently affected (Tables S3B, S4). For example, a gene cluster (PPA0582-0585), with homology to the arginine deiminase (ADI) pathway was 5- to 7-fold upregulated in the EP (Figures 3 and 4). This pathway catalyzes the conversion of arginine to ornithine via citrulline, employing the enzymes arginine deiminase (PPA0583), ornithine carbamoyltransferase (PPA584) and carbamate kinase (PPA0585), thereby generating NH_3_ and ATP. There is also an arginine/ornithine antiporter (PPA0582) encoded in this cluster. The arginine repressor ArgR is encoded just downstream (PPA0586). This system could be a powerful way for *P. acne*s to counteract the acidification of the culture medium in the course of propionate formation, akin to *Streptococcus suis* and sourdough lactic acid bacteria, which use the ADI pathway to facilitate survival in acidic conditions [32]. The ADI pathway has also been reported to function as an additional energy conserving pathway during anaerobic growth of *Bacillus licheniformis* and *Pseudomonas aeruginosa* [33],[34].

By contrast, genes involved in arginine biosynthesis such as argininosuccinate synthase (PPA2201), converting aspartate and citrulline into argininosuccinate, and the cluster PPA1347-1350 were upregulated in the SP (Figure 3). The latter encodes enzymes catalyzing the conversion of glutamate to ornithine. Other genes involved in amino acid metabolism and upregulated in the SP were two copies of a glutamine synthetase (PPA0664, PPA0671, 30% protein sequence identity), which converts glutamate into glutamine, and the cluster PPA0997-1001, encoding a system to interconvert glutamine and (dihydro)orotate. In conclusion, marked deregulation of enzymes linked to the urea cycle can be detected between the EP and the SP.

### Deregulation of genes involved in heat shock and other stress responses

Heat shock proteins have been implicated in the virulence of *P. acnes*, for example in the induction of a proinflammatory response in keratinocytes [29], [35]. The genes encoding cytosolic 70-kDa heat-shock protein (Hsp70) chaperones were highly expressed in both growth phases (DnaK, PPA2040; DnaJ, PPA2038; GrpE, PPA2039); the same is true for the trigger factor (PPA1575), a protein with peptidyl-prolyl *cis-trans* isomerase activity, and the first protein to encounter nascent polypeptide chains as they emerge from the ribosome. By contrast, the chaperonins GroEL (two genes: PPA0453 and PPA1772, 65% protein sequence identity) and GroES (PPA1773) were deregulated between the growth phases, i.e. 6 to 11-fold upregulated in the EP (Table S4). The RNA levels of these three genes exhibited the strongest growth phase dependency among all genes. GroEL of *P. acnes* has been implicated in the stimulation of cytokine production, such as interleukin (IL)-1-alpha, tumor necrosis factor (TNF)-alpha or granulocyte/macrophage colony-stimulating factor (GM-CSF) in keratinocytes [35].

In the SP 7.5-fold upregulation of *hsp20* (PPA0737, 18kDa antigen 2) was detected; this gene encodes an alpha-crystallin-like protein homologous to HspX (Acr, Rv2031c) of *Mycobacterium tuberculosis* [36]; HspX is an immunodominant antigen that is recognized by the majority of patients with active tuberculosis. In addition, other genes related to the heat shock response were upregulated in the SP, including those encoding a DnaJ homolog (PPA0916), a DnaK family protein (PPA1098), a PAC2 (proteasome assembly chaperone) family protein (PPA1099), and three Hsp100 chaperones (ClpB (PPA2021), ClpC (PPA0278) and ClpX (PPA1571)).

Several other genes that encode proteins related to stress responses and defense were also induced in the SP, for example PPA0580 and PPA0581. These are clustered directly upstream of the highly induced EP genes PPA0582-0585, which encode the ADI pathway. PPA0580 is an osmotically inducible (OsmC)-like protein. OsmC-family proteins apparently function as peroxiredoxins [37]. PPA0581 encodes a protein with homology to Hsp31 of *E. coli*, which has chaperone activity [38]. Interestingly, PPA0580 and PPA0581 were the most strongly deregulated genes in all microarray experiments (Figure 4).

Upregulation of the genes PPA1449 (HicB-like protein) and PPA1450 (hypothetical *P. acnes* specific protein) was also a characteristic of the SP. HicB is part of a cluster, HicAB, which is predicted to be a RNA-targeting toxin-antitoxin (TA) system present in a variety of bacteria [39]; *hicAB* transcription in *E. coli* was shown to be strongly induced by nutrient starvation [40]. An additional, larger SP gene cluster is PPA0387-0394, which includes a protein similar to alkaline shock proteins (PPA0387), and RpoE (PPA0392), encoding the stress response sigma factor σ^24^. Other gene clusters upregulated in the SP were: PPA1973-1975, encoding a nitric-oxide (NO) reductase (NorB, PPA1975), which could function as an NO detoxifying system to eliminate NO produced by the host immune system; PPA1548-1550, encoding proteins similar to SufB (PPA1549) and SufD (PPA1548), which are part of the iron-sulfur cluster scaffold complex SufBCD, which functions under iron starvation and oxidative stress conditions [41].

### Growth phase-dependent expression of putative virulence factors

Two machineries for protein secretion, the Sec-signal recognition particle (Sec-SRP) and the twin arginine translocation (Tat) systems, were strongly expressed by *P. acnes*, as shown by the expression levels of *secF* (PPA1163), *secE* (PPA1892), *yajC* (PPA1161), *tatB* (PPA0643) and *tatC* (PPA1378). The Sec-SRP system, but not the Tat system, was more strongly expressed during the EP than the SP, in particular the components *secA* (PPA1333), *secF* (PPA1163) and *ftsY* (PPA1447) and also signal peptidase I (PPA1434). Previously, we identified secreted factors of *P. acnes* by a proteomic approach [26]. Strikingly, we found that not only were components of the Sec-SRP and Tat systems strongly expressed but also secreted factors, most of which were upregulated during the EP; these encode endo-glycoceramidase (PPA0644), p60 (PPA0721), lysophospholipase (PPA2142), rare lipoprotein A (PPA2175), GroEL (PPA1772) and several hypothetical proteins (PPA0532-534, PPA2141, PPA2239). Regarding the CAMP factors, the genes camp1, 2 and 4 had significant expression levels; camp4 (PPA1231) ranked among the most strongly expressed genes, independent of the growth phase. camp1 (PPA1340) was preferentially expressed in the EP, as was camp2 (PPA0687). CAMP2 and 4 have been shown to be secreted proteins, and CAMP1 is both secreted and surface-associated [26], [42]. By contrast, increased expression of genes encoding secreted factors in the SP was scarce, with the exception of genes encoding a hyaluronidase (PPA0380) and the immunoreactive adhesin PPA2127 (PA-25957). Albeit highly expressed, PPA2127 is not translated in strain KPA, possibly due to genetic mutations [25], [26].

### Conclusions

*P. acnes* is a life-long inhabitant of the skin of most human bodies. As such, this organism must have developed efficient strategies to adapt to the microenvironment within sebaceous follicles. The transcriptome of *P. acnes* as reported here provides valuable insights into the lifestyle of this skin commensal. The data indicate that the organism has several metabolic options in addition to propionic acid fermentation. Energy conservation employs substrate-level phosphorylation (e.g. ADI pathway) as well as respiration, utilizing fumarate and possibly also nitrate as terminal electron acceptors. Given its persistence in sebaceous follicles of the human skin, the question arises as to how *P. acnes* adapts its metabolic profile in the face of altered sebaceous gland activity during puberty, when androgen-dependent stimulation of gland secretion affects oxygen and nutrient (i.e. sebum) levels in the follicular microenvironment. Our data indicate that *P. acnes* is able to carry out aerobic respiration, thereby employing alternative terminal oxidases. It is not apparent why only nanoaerophilic conditions but not higher oxygen tensions are favorable for growth; the organism possesses the important antioxidant defense systems, and employs a set of additional stress response systems, whose functions have not been studied in this organism.

Cell culture experiments showed that host responses to *P. acnes* varied depending on the bacterial strain used [10], [11], [12]. We also noticed in our previous work substantial differences between *P. acnes* strains with respect to their effects on epithelial prostate cells [28]: strain 266 (I-1a, ST18) infection of RWPE1 cells led to significant deregulation of 260 host cell genes, the majority of which were inflammation-associated genes, whereas the KPA strain (I-2, ST34) impacted on only 97 gene transcripts (fold-change >3 or <-3), indicating that strain 266 possesses a higher immunostimulatory activity than KPA. In the present work, we investigated the basis for differences between strains KPA and 266 on genomic and transcriptional levels. Genome comparison cannot easily explain the phenotypic differences between these two strains regarding their inflammatory potential; the genome of strain 266 does not contain additional known or putative virulence factors, which are correspondingly absent from strain KPA. Instead, several genomic regions were identified that encode KPA-specific functions; these regions differed also among the major phylotypes of *P. acnes*. They were actively transcribed in strain KPA, indicating their functionality, and encode fitness functions such as additional defense systems against other (Gram-positive) bacteria and additional nutrient uptake and degradation systems.

A possible explanation for the elevated inflammatory potential of strain 266 compared to strain KPA could be deduced from the expression profiles of a number of predicted or characterized host-interacting factors of *P. acnes*. For instance, the gene encoding the lipase GehA is upregulated in strain 266; GehA is suspected to hydrolyze sebum triacylglycerides, resulting in the release of glycerol and free fatty acids [24], [43]. Released fatty acids are thought to be inflammatory; they favor ductal hypercornification and increase adhesion between *P. acnes* and cells of the hair follicle, promoting colonization of *P. acnes* [43]. Another example is the upregulation of the *htaA* gene in strain 266; HtaA (PA-4687) is an iron acquisition protein, which has been shown to be strongly immunoreactive [25].

Besides phylotype-specific variation, some properties of *P. acnes*, e.g. biofilm formation, are apparently independent of the phylotype [9]. We suspect that strain differences between members of the same phylotype are, at least in part, due to phase variation, e.g. affecting the expression of the dermatan-sulphate adhesins PPA2127 and PPA2210; however, it is likely that other factors are also important.

Taken together, our data support the assumption that the severity of *acne vulgaris* and other *P. acnes*-associated diseases depends upon the respective phylotype of the causative strain. An opportunistic scenario for the formation of inflammatory acne could thus be envisaged: (i) the presence of a *P. acnes* strain with elevated virulence potential, preferably a member of the phlyogenetic subdivision I-1a expressing phase-variable adhesins, and (ii) favorable conditions for its active growth, i.e. nutrient (e.g. sebum) availability and anaerobic or nanoaerophilic conditions after comedo formation, and (iii) a predisposed host with an unbalanced immunological response to *P. acnes*.

### Note

A recent publication by McDowell et al (Microbiology, 2011 Apr 21) presented an alternative MLST typing scheme for *P. acnes*. According to this scheme the strains in our study are classified as follows: KPA, type IB_1_ (ST10); 266, type IA-CC6 (ST25); SK137, type IA-CC6 (ST11); SK187, type IA (ST39) and J139, type II (ST40).

## Materials and Methods

### Strains and growth conditions

*P. acnes* strain 266 was chosen for sequencing; it is a type I-1a/ST18 strain, which was isolated from a pleuropulmonary infection (kindly provided by Oliver Knapp and Michel Popoff, Pasteur Institute, Paris, France). For transcriptome experiments strain 266 and KPA171202 (I-2/ST34) were used; the genome of the latter strain was previously sequenced [15]. 14 additional *P. acnes* strains, listed in table 2, were used for PCR detection of four large genomic regions. The phylotypes of these human isolates were previously identified using the MLST scheme and database (http://pacnes.mlst.net) developed by Lomholt and Kilian [14]. All *P. acnes* strains were cultured on Brucella agar plates under anaerobic conditions at 37°C for three days. For liquid cultures, plate-grown bacteria were resuspended and washed in brain heart infusion (BHI) broth (Sigma-Aldrich); BHI broth was inoculated with *P. acnes* (OD_600_ 0.01) and cultures were grown to mid exponential (OD_600_ 0.3), late exponential (OD_600_ 0.6) or stationary phases (OD_600_ 0.8) at 37°C under anaerobic conditions using the Gas-Pak™ system (Oxoid). To avoid inconsistent growth conditions, all reported growth experiments were performed in BHI medium from the same batch. In addition, for comparative experiments (strain 266 versus strain KPA, exponential phase versus stationary growth phase) the culture medium was taken from the same autoclaved liquid medium stock. To avoid variable anaerobic conditions in comparative experiments due to the usage of the Gas-Pak system, *P. acnes* strains were cultured in the same GasPak container, guaranteeing identical growth conditions.

### DNA isolation and PCR

DNA from ***P. acnes*** was isolated using the MasterPure™ Gram Positive DNA Purification Kit (Epicentre). For the PCR detection of four large genomic regions in *P. acnes* the primers listed below were used to amplify 400–500 bps of the corresponding genomic region (two primer pairs for each region). PCR amplifications were done using Platinum ***Taq*** DNA polymerase (Invitrogen). Samples were initially heated at 95°C for 3 min, followed by 30 cycles consisting of 1 min at 95°C, 30 s at 55°C, and 45 s at 72°C. IS1_PPA0849_for ACACACCGGCGTCACTTTCA, IS1_PPA0849_rev ATGTTGAGGGCGGTGACGTT; IS1_PPA0860_for CCGCTATTCGCAATCGCTTC, IS1_PPA0860_rev GAGAGCCGGAACCGAGAACA; IS2_PPA1280_for GGATCGGGTCGTCTTGTTGG, IS2_PPA1280_rev CAGCAACCTGGGCGAACTCT; IS2_PPA1288_for GCAGCGGTCTCTGACCAACA, IS2_PPA1288_rev TCCCCGAGAGCCAATCAGAG; IS3_PPA1585_for CGTGACTGGCTGCTGCATCT, IS3_PPA1585_rev CGTTGCCGTCCAAGTGTTTG; IS3_PPA1612_for GCCGCCCTAGGCAAGAAACT, IS3_PPA1612_rev GCAGGCGAGCAAGCTGGTAT; IS4_PPA2056_for CGACATTTCGCGGGACTACC, IS4_PPA2056_rev ACGACCTCGTGCTTCCCAAC; IS4_PPA2070_for TACTGCGCCGCTCAACTCAC, IS4_PPA2070_rev CATCGTGCACCCTCATCCAC.

### Genome sequencing, assembly and gap closure

A pyrosequencing approach was used for whole-genome sequencing of *P. acnes* strain 266. Chromosomal DNA was nebulized, a single-stranded template DNA library prepared and sequenced using a Genome Sequencer FLX Instrument and Titanium chemistry (Roche Applied Science); the library preparation and DNA sequencing were performed according to manufacturer's protocols (Roche Applied Science). The sequence data (247,277 reads) were assembled using the Newbler Assembler (454 Life Sciences). 15 contigs were obtained (>500 bases); a 36-fold coverage was obtained. Editing of sequences was done with the GAP4 software as part of the Staden package [44]. To solve problems with misassembled regions caused by repetitive sequences and to close remaining sequence gaps, standard PCR, combinatorial multiplex PCR, and primer walking on recombinant plasmids were applied. The genome sequence reported in this paper has been deposited in the GenBank database with accession number CP002409.

### Bioinformatics

Coding sequences (CDS) and open reading frames (ORFs) were predicted with YACOP [45], using the ORF finders Glimmer, Critica and Z-curve. The output was verified and edited manually using criteria such as the presence of a ribosome binding site, GC frame plot analysis, and similarity to known protein-encoding sequences. Annotation was done in a two-step approach. Initially, all proteins were screened against Swiss-Prot data and publicly available protein sequences from other completed genomes. By protein sequence comparison with the Pfam, GenBank, ProDom, COG and Prosite databases, all predictions were verified or modified manually using the ERGO software package [46], licensed by Integrated Genomics™. Complete genome comparisons were done with a protein sequence-based bidirectional BLAST approach. For genome-wide comparisons of nucleotide sequences the Artemis Comparison Tool (ACT) was used [47]. Sequence homologies were only mentioned in this study for proteins with an amino acid identity of >25% and an overlap of the query and subject sequence of >90%. To identify genomic islands the Island Viewer was used, a computational tool that integrates three different genomic island prediction methods, i.e. IslandPick, IslandPath-DIMOB, and SIGI-HMM [48]. For the analysis and mapping of pathways of *P. acnes* KEGG and KEGG MAPPER were used (http://www.genome.jp/kegg/); several pathways were manually curated.

### RNA isolation, quantification and quality control

Total RNA was isolated by a modified TRIzol® reagent RNA preparation method (Invitrogen). Briefly, a *P. acnes* culture was harvested by centrifugation and the pellet (<10^9^ bacteria) was resuspended in 1 ml TRIzol. The resuspension was added to a tube with glass beads (Lysing Matrix B, MP Biochemicals), and processed twice for 25 s at speed 6.5 in a FastPrep FP120 shaker (Savant). After centrifugation (5 min, 8000×*g*) the upper phase was transferred to a microcentrifuge tube and mixed with 200 µl chloroform. Subsequently, the steps as detailed in the manufacturer's protocol for the TRIzol® reagent were carried out (Invitrogen). All centrifugation steps were done at 4°C. The amount of RNA was determined by OD_260_/OD_280_ measurement using a NanoDrop® 1000 spectrophotometer (Kisker). The RNA size, integrity and the amount of total RNA were measured with a Bioanalyzer 2100 (Agilent Technologies) with a RNA Nano 6000 microfluidics kit.

### Microarray design

*P. acnes* KPA171202 microarrays were designed with OligoWiz [49] as 4x44K custom microarrays including all ORFs and intergenic regions (IGR) from the whole genome of *P. acnes* KPA171202. ORF specific probes were designed as sense oligonucleotides of the coding strand. IGR, 3′- and 5′-overlapping probes to ORFs were designed as sense and reverse complement probes to the +-strand. Overlapping probes to ORFs were generated by sequence stretches comprising -45 bases downstream or +45 bases upstream and the first or last 45 bases of the ORF, respectively. An oligo aim length of 60 bases with a minimal length of 50 bases was used together with max homology (avoid self hit) 97, 80% cross hybridization maximum length (avoid self hit) and a random primed position preference score within OligoWiz. Dependent on these parameters and the sequence input length, each ORF and IGR was covered by several specific oligonucleotide probes. In total 39,094 specific *P. acnes* KPA171202 probe sequences were uploaded to eArray (Agilent Technologies, https://earray.chem.agilent.com/earray/) as ChIP application with a customer specified (ordered according chromosomal location) feature layout.

For comparative expression profiling of the two *P. acnes* strains 266 and KPA, all oligomers of the microarray were identified by BLASTN which matched 100% between both genomes; only those 29,587 oligomers (75.7%) were used to calculate expression differences between the two *P. acnes* strains.

### Microarray expression profiling

Microarray experiments were performed as dual-color hybridizations. In order to compensate specific effects of the dyes and to ensure statistically relevant data analysis, a color-swap dye-reversal was performed [50]. RNA labeling was performed with the two color Quick Amp Labeling Kit (Agilent Technologies) using FullSpectrum MultiStart Primer for T7 IVT RNA Amplification (BioCat GmbH) as random T7 labeling. In brief, mRNA was reverse transcribed and amplified using a FullSpectrum MultiStart-T7-promotor primer and was labeled either with Cyanine 3-CTP or Cyanine 5-CTP. Alternatively, the total RNA samples were amplified with the TransPlex Whole Transcriptome Amplification Kit (Sigma-Aldrich) and labeled with BioPrime Plus Array CGH Indirect Genomic Labeling System (Invitrogen) as WTA BioPrime indirect. In brief, a library was generated by stand displacement reaction using phi-29 polymerase and quasi-random primers followed by 17 PCR cycles. The resultant cDNA library was labeled with Klenow polymerase in an indirect reaction using animoallyl nucleotides and subsequent chemical NHS-ester Cy-dye coupling. After precipitation, purification and quantification, labeled samples (cRNA for random T7 labeling or cDNA for WTA BioPrime indirect labeling) were hybridized separately to the 4x44K custom-commercial microarrays according to the supplier's protocol (Agilent Technologies). Scanning of microarrays was performed with 5 µm resolution and extended mode using a high resolution microarray laser scanner (G2505, Agilent Technologies). Raw microarray image data were extracted and analyzed with the Image Analysis/Feature Extraction software G2567AA (Version A.10.5.1.1, Agilent Technologies). The extracted MAGE-ML files were further analyzed on reporter and sequence levels with the Rosetta Resolver Biosoftware, Build 7.2.2.0 SP1.31. Ratio profiles comprising single hybridizations were combined in an error-weighted fashion to create ratio experiments. A 2–fold change expression cut-off for ratio experiments was applied together with anti-correlation of ratio profiles rendering the microarray analysis highly significant (P-value >0.01), robust and reproducible. Additionally, data derived from the different labeling procedures (random T7 labeling and WTA BioPrime indirect labeling) were compared and combined. Only results derived from both labeling methods were considered as relevant for further analysis. The data presented in this publication have been deposited in NCBIs Gene Expression Omnibus (GEO, http://www.ncbi.nlm.nih.gov/geo/) and are accessible through GEO Series accession number GSE26738.

In addition, the microarray results can be visualized at http://bioinfo.mpiib-berlin.mpg.de/pacnes/. All oligomers are plotted according to their expression levels (absolute intensities as well as log-ratios/fold-changes). Genomic deletions in strain 266 are marked as yellow boxes. For a full legend see: http://bioinfo.mpiib-berlin.mpg.de/pacnes/icons/legend.png.

## Supporting Information

Figure S1 **Genomic islands of sequenced Propionibacteria genomes.** The meta-program Island viewer (http://www.pathogenomics.sfu.ca/islandviewer/query.php) was used to predict genomic islands. Predicted genomic islands are colored within the circular image based on the following tools: SIGI-HMM, orange; IslandPath-DIMOB, blue; integrated, red. Black line plot: GC content (%). The numbers and letters of the islands correspond to figure 1 and table S1. Strain-specific genomic regions (listed in table S1) often, but not always, correspond to the predicted islands. The island 1′ in the genome of strain SK137 is dissimilar to the island 1 of strain KPA, see table S1.
(DOC)

Table S1 **Genomic differences between** ***P. acnes*** **strains.** Listed are all regions > 2 genes which differ (deletions, insertions, replacements, differences) between KPA and 266 (A), KPA and SK137 (B), and 266 and SK137 (C). In brackets: size of each genomic region (in kb). Color code: green, specific regions in KPA; yellow, specific regions in 266; red, specific regions in SK137; grey, present in both genomes, but with substantial differences (identity <50% or frameshifted). The numbers and letters in the first column correspond to the genomic regions depicted in figures 1 and S1.
(DOC)

Table S2 **Gene expression changes between two** ***P. acnes*** **strains grown anaerobically to (A) mid-exponential phase or to (B) late-exponential phase in BHI medium.** The data shown is based only on oligomers of the microarray which matched 100% in the two genomes KPA and 266. The intensities were calculated as the average intensity from oligomers that covered the respective gene. Only significantly de-regulated genes with fold changes >2 or <-2 are listed. For further details, see the GEO entry GSE26738 or go to: http://bioinfo.mpiib-berlin.mpg.de/pacnes/.
(XLS)

Table S3 **A: Biological processes associated to genes differentially regulated in the** ***P. acnes*** **strains KPA and 266.** All 119 and 316 genes (see Table S2) that were de-regulated (fold-change of >2 or <-2) in the two strains grown to mid-exponential and late exponential growth phases, respectively, were considered in this analysis. In the exponential growth phase (OD 0.3), 82 genes were up-regulated in strain 266, 40 of which (49%) were KEGG-mappable; in KPA, 37 were up-regulated, only 7 (19%) were KEGG-mappable. In the late exponential growth phase (OD 0.6), 194 genes were up-regulated in strain 266, 74 of which (38%) were KEGG-mappable; in KPA, 122 genes were up-regulated, 57 of which (46%) were KEGG-mappable. KEGG MAPPER (http://www.genome.jp/kegg/mapper.html) was used to map the corresponding genes to biological processes. Only processes with at least 2 hits are listed. **B: Biological processes associated to genes up-regulated in** ***P. acnes*** **KPA grown to exponential or stationary growth phase.** All genes with a fold-change of >2 or <-2 were considered in this analysis. 137 genes were exponential phase (EP) genes, 78 of which (57%) were KEGG-mappable. 122 genes were stationary phase (SP) genes; 38 of which (31%) were KEGG-mappable. Only processes with at least 2 hits are listed.
(DOC)

Table S4 **Gene expression changes between exponential and stationary phase** ***P. acnes*** **KPA171202.** *P. acnes* was grown in BHI medium to OD 0.3 (exp) and OD 0.8 (stat) under anaerobic conditions. The intensities were calculated as the average intensity of all oligomers that covered the respective gene. Only significantly de-regulated genes with fold changes >2 or <-2 are listed. For further details see the GEO entry GSE26738 or go to: http://bioinfo.mpiib-berlin.mpg.de/pacnes/.
(XLS)
