# Supplementary material for: Comparative Genomics and Transcriptomics of Propionibacterium acnes
Source: PLoS One. 2011 Jun 27;6(6):e21581. doi: 10.1371/journal.pone.0021581 (PMC3124536; doi:10.1371/journal.pone.0021581)
Supplement: Figure S1 — Genomic islands of sequenced Propionibacteria genomes. The meta-program Island viewer (http://www.pathogenomics.sfu.ca/islandviewer/query.php) was used to predict genomic islands. Predicted genomic islands are colored within the circular image based on the following tools: SIGI-HMM, orange; IslandPath-DIMOB, blue; integrated, red. Black line plot: GC content (%). The numbers and letters of the islands correspond to figure 1 and table S1. Strain-specific genomic regions (listed in table S1) often, but not always, correspond to the predicted islands. The island 1′ in the genome of strain SK137 is dissimilar to the island 1 of strain KPA, see table S1. (DOC) [file pone.0021581.s001.doc]

**Figure S1. Genomic islands of sequenced Propionibacteria genomes.**

The meta-program Island viewer (http://www.pathogenomics.sfu.ca/islandviewer/query.php) was used to predict genomic islands. Predicted genomic islands are colored within the circular image based on the following tools: SIGI-HMM, orange; IslandPath-DIMOB, blue; integrated, red. Black line plot: GC content (%). The numbers and letters of the islands correspond to figure 1 and table S1. Strain-specific genomic regions (listed in table S1) often, but not always, correspond to the predicted islands. The island 1’ in the genome of strain SK137 is dissimilar to the island 1 of strain KPA, see table S1.

*P. acnes* KPA (I-2/ST34) *P. acnes* 266 (I-1a/ST18)


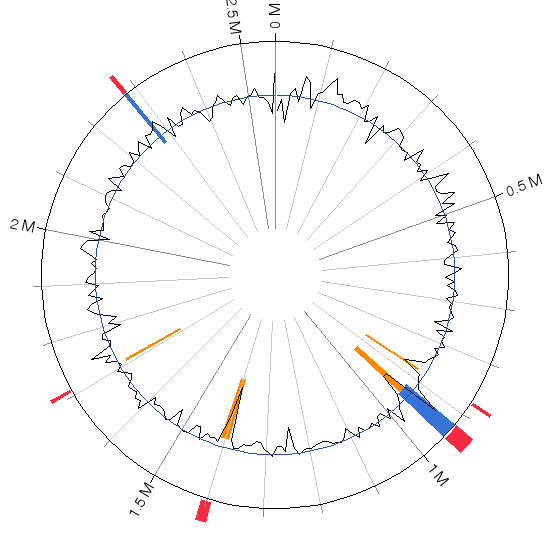

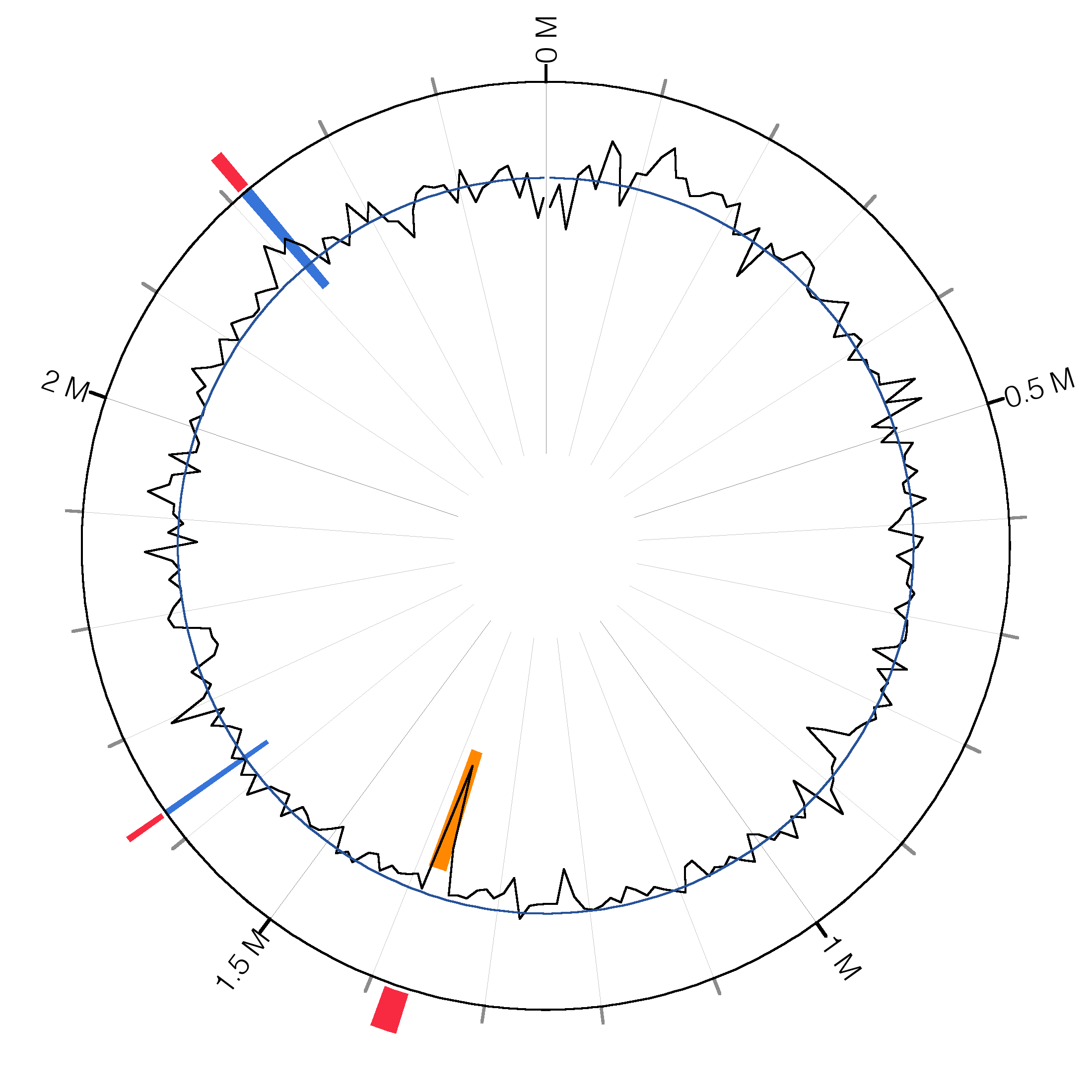


**2**

**4**

**1**

**3**

**2**

**4**

*P. acnes* SK137 (I-1a/new ST) *P. freudenreichii* ssp. *shermanii*
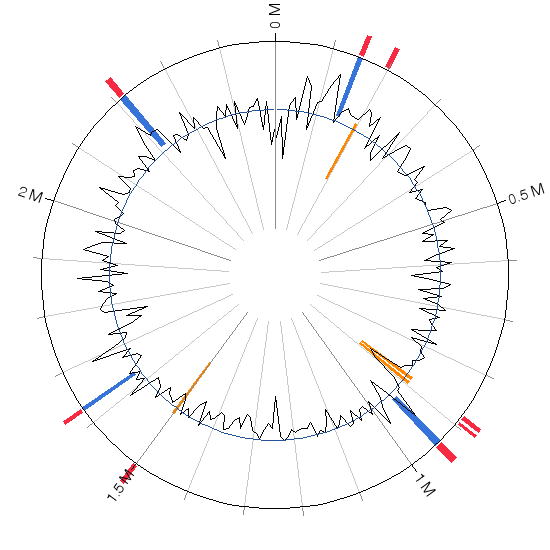

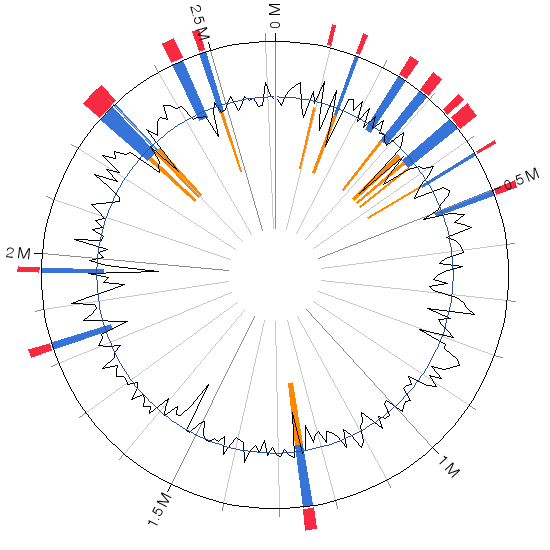


**1‘**

**4**

**B**

**A**
