# Supplementary material for: Comparative Genomics and Transcriptomics of Propionibacterium acnes
Source: PLoS One. 2011 Jun 27;6(6):e21581. doi: 10.1371/journal.pone.0021581 (PMC3124536; doi:10.1371/journal.pone.0021581)
Supplement: Table S1 — Genomic differences between P. acnes strains. Listed are all regions > 2 genes which differ (deletions, insertions, replacements, differences) between KPA and 266 (A), KPA and SK137 (B), and 266 and SK137 (C). In brackets: size of each genomic region (in kb). Color code: green, specific regions in KPA; yellow, specific regions in 266; red, specific regions in SK137; grey, present in both genomes, but with substantial differences (identity <50% or frameshifted). The numbers and letters in the first column correspond to the genomic regions depicted in figures 1 and S1. (DOC) [file pone.0021581.s002.doc]

**Table S1: Genomic differences between *P. acnes* strains**

Listed are all regions > 2 genes which differ (deletions, insertions, replacements, differences) between KPA and 266 (A), KPA and SK137 (B), and 266 and SK137 (C). In brackets: size of each genomic region (in kb). Color code: green, specific regions in KPA; yellow, specific regions in 266; red, specific regions in SK137; grey, present in both genomes, but with substantial differences (identity <50% or frameshifted). The numbers and letters in the first column correspond to the genomic regions depicted in figures 1 and S1.

A) KPA versus 266

| **region** | **genomic**  **position in kb** | **KPA genes** | **266 genes** | **Prominent predicted function(s)**  green, in KPA; yellow, in 266; grey, in both but dissimilar |
| --- | --- | --- | --- | --- |
| 5 | 345.4 | PPA0295-0299 (4.6) | - | 4-hydroxythreonine-4-phosphate dehydrogenase 2 |
| 6 | 420.2 | PPA0372-0382 (10.8) | PAZ_c03880-PAZ_c03950 (6.4) | 3-ketoacyl-(acyl-carrier-protein) reductase, glycosyl hydrolase hyaluronate lyase |
|  | 809.6 | PPA0738-0740 (2) | - | gluconokinase, gluconate permease |
| **1** | 924.0 | PPA0844-0874 (28.5) | PAZ_c08910-PAZ_c09070 (11.6) | Tra-region, thiopeptide biosynthesis, phosphate regulon sensor protein phoR, ABC transport system |
| 8 | 1579.0 | - | PAZ_c15380-PAZ_c15460 (8.8) | peptide ABC transport system,  glycosyl hydrolases |
| **3** | 1708.0 | PPA1578-1613 (32) | - | phage-related proteins, putative type III restriction enzyme |
|  | 1817.4 | PPA1663-1666 (2.8) | PAZ_c17290-PAZ_c17320 (3) | thrombospondin type 3 repeat protein, putative adhesin |
| 7 | 1984.7 | PPA1820/1821 (5) | - | beta-galactosidase fused to beta-N-acetylhexosaminidase, putative sialidase |
|  | 2067.0 | PPA1906 (2) | PAZ_c19850/  PAZ_c19860 (2) | thrombospondin type 3 repeat protein |
| 9 | 2104.0 | PPA1944 (2.4) | PAZ_c20210-PAZ_c20270 (8.6) | ABC transporter |
|  | 2154.3 | PPA1983/PPA1984 (2.4) | PAZ_c20720-PAZ_c20740 (2.4) | thrombospondin type 3 repeat protein |
| **4** | 2232.8 | PPA2055-2092 (34) | PAZ_c21490-PAZ_c21630 (13.6) | beta-glucanase, alpha-galactosidase, alpha-L-fucosidase precursor, transporter,  ABC transporter |

B) KPA versus SK137

| **region** | **genomic**  **position in kb** | **KPA genes** | **SK137 genes** | **Prominent predicted function(s)**  green, in KPA; red, in SK137; grey, in both but dissimilar |
| --- | --- | --- | --- | --- |
| A | 145.2 | - | HMPREF0675_3127-3133 (6.5) | N-acetylmuramoyl-L-alanine amidase, recombinase, ABC transporter |
| B | 185.0 | - | HMPREF0675_3169-3193 (20.8) | CobQ/CobB/MinD/ParA nucleotide binding domain protein, streptolysin biosynthesis genes (SagBCD), ABC transporter (MtrAB homolog) |
| 5 | 345.4 | PPA0295-0299 (4.6) | - | 4-hydroxythreonine-4-phosphate dehydrogenase 2 |
| 6 | 420.2 | PPA0372-0382 (10.8) | HMPREF0675_3412-3416 (6.5) | 3-ketoacyl-(acyl-carrier-protein) reductase  hyaluronate lyase |
|  | 809.6 | PPA0738-0740 (2) | - | gluconokinase,  gluconate permease |
| **1** | 924.0 | PPA0844-0874 (28.5) | HMPREF0675_3911-3930 (13) | Tra-region, thiopeptide biosynthesis, histidine kinase, ABC transport system |
| **2** | 1386.0 | PPA2384-1305 (28.6) | - (2.4) | NRPS system |
| 8 | 1579.0 | - | HMPREF0675_4518-4526 (9) | peptide ABC transport system |
| **3** | 1708.0 | PPA1578-1613 (32) | - | phage-related proteins, putative type III restriction enzyme |
|  | 1817.4 | PPA1663-1666 (2.8) | HMPREF0675_4712-4714 (2.5) | thrombospondin type 3 repeat protein, putative adhesin |
| 7 | 1984.7 | PPA1820/1821 (5) | - | beta-galactosidase fused to beta-N-acetylhexosaminidase, putative sialidase |
|  | 2067.0 | PPA1906 (2) | HMPREF0675_4963/  4964 (2) | thrombospondin type 3 repeat protein |
| 9 | 2104.0 | PPA1944 (2.4) | HMPREF0675_4999-5004 (8.6) | ABC transporter |
|  | 2154.3 | PPA1983/PPA1984 (2.4) | HMPREF0675_5048-5050 (2.4) | thrombospondin type 3 repeat protein |
| **4** | 2232.8 | PPA2055-2092 (34) | HMPREF0675_5127-5143 (13.6) | beta-glucanase, alpha-galactosidase, alpha-L-fucosidase precursor, transporter  ABC transporter |

C) 266 versus SK137

| **region** | **genomic**  **position in kb** | **266 genes** | **SK137 genes** | **Prominent predicted function(s)**  yellow, in 266 (and KPA); red, in SK137 |
| --- | --- | --- | --- | --- |
|  | 146.0 | - | HMPREF0675_3127-3133 (6.5) | N-acetylmuramoyl-L-alanine amidase, recombinase, ABC transporter |
|  | 185.6 | - | HMPREF0675_3169-3193 (20.8) | CobQ/CobB/MinD/ParA nucleotide binding domain protein, streptolysin biosynthesis genes (SagBCD), ABC transporter (MtrAB homolog) |
| **2** | 1361.8 | PAZ_c13310-PAZ_c13670 (28.6) | - | Non-ribosomal peptide synthetase (NRPS) system |
