# Supplementary material for: Comparative Genomics and Transcriptomics of Propionibacterium acnes
Source: PLoS One. 2011 Jun 27;6(6):e21581. doi: 10.1371/journal.pone.0021581 (PMC3124536; doi:10.1371/journal.pone.0021581)
Supplement: Table S3 — A: Biological processes associated to genes differentially regulated in the P. acnes strains KPA and 266. All 119 and 316 genes (see Table S2) that were de-regulated (fold-change of >2 or <-2) in the two strains grown to mid-exponential and late exponential growth phases, respectively, were considered in this analysis. In the exponential growth phase (OD 0.3), 82 genes were up-regulated in strain 266, 40 of which (49%) were KEGG-mappable; in KPA, 37 were up-regulated, only 7 (19%) were KEGG-mappable. In the late exponential growth phase (OD 0.6), 194 genes were up-regulated in strain 266, 74 of which (38%) were KEGG-mappable; in KPA, 122 genes were up-regulated, 57 of which (46%) were KEGG-mappable. KEGG MAPPER (http://www.genome.jp/kegg/mapper.html) was used to map the corresponding genes to biological processes. Only processes with at least 2 hits are listed. B: Biological processes associated to genes up-regulated in P. acnes KPA grown to exponential or stationary growth phase. All genes with a fold-change of >2 or <-2 were considered in this analysis. 137 genes were exponential phase (EP) genes, 78 of which (57%) were KEGG-mappable. 122 genes were stationary phase (SP) genes; 38 of which (31%) were KEGG-mappable. Only processes with at least 2 hits are listed. (DOC) [file pone.0021581.s004.doc]

**Table S3A: Biological processes associated to genes differentially regulated in the *P. acnes* strains KPA and 266**

All 119 and 316 genes (see Table S2) that were de-regulated (fold-change of >2 or <-2) in the two strains grown to mid-exponential and late exponential growth phases, respectively, were considered in this analysis. In the exponential growth phase (OD 0.3), 82 genes were up-regulated in strain 266, 40 of which (49%) were KEGG-mappable; in KPA, 37 were up-regulated, only 7 (19%) were KEGG-mappable. In the late exponential growth phase (OD 0.6), 194 genes were up-regulated in strain 266, 74 of which (38%) were KEGG-mappable; in KPA, 122 genes were up-regulated, 57 of which (46%) were KEGG-mappable. KEGG MAPPER (http://www.genome.jp/kegg/ mapper.html) was used to map the corresponding genes to biological processes. Only processes with at least 2 hits are listed.

| **KEGG**  **map *** | **Biological process** | **266**  **(OD 0.3)** | **266**  **(OD 0.6)** | **KPA**  **(OD 0.3)** | **KPA**  **(OD 0.6)** |
| --- | --- | --- | --- | --- | --- |
| pac03010 | Ribosome | 20 | - | - | 3 |
| pac00190 | Oxidative phosphorylation | - | - | - | 17 |
| pac00330 | Arginine and proline metabolism | 2 | 9 | - | 4 |
| pac02010 | ABC transporters | 2 | 8 | - | 5 |
| pac00230 | Purine metabolism | - | 8 | - | 7 |
| pac00970 | Aminoacyl-tRNA biosynthesis | 3 | 8 | - | - |
| pac00240 | Pyrimidine metabolism | - | 7 | - | 5 |
| pac00300 | Lysine biosynthesis | - | 7 | - | 2 |
| pac00620 | Pyruvate metabolism | 2 | 5 | - | - |
| pac00010 | Glycolysis / Gluconeogenesis | - | 4 | - | 3 |
| pac02020 | Two-component system | - | 3 | - | 4 |
| pac00250 | Alanine, aspartate and glutamate metabolism | - | 6 | - | - |
| pac00550 | Peptidoglycan biosynthesis | - | - | - | 5 |
| pac00270 | Cysteine and methionine metabolism | - | 4 | - | - |
| pac00520 | Amino sugar and nucleotide sugar metabolism | - | 3 | - | 3 |
| pac00020 | Citrate cycle (TCA cycle) | - | 3 | - | 3 |
| pac00910 | Nitrogen metabolism | - | - | - | 5 |
| pac00052 | Galactose metabolism | - | 2 | - | 3 |
| pac00640 | Propanoate metabolism | 2 | 3 | - | - |
| pac00650 | Butanoate metabolism | - | 2 | - | 3 |
| pac03440 | Homologous recombination | - | 4 | - | - |
| pac00750 | Vitamin B6 metabolism | - | 4 | - | - |
| pac00051 | Fructose and mannose metabolism | 2 | 2 | - | - |
| pac02060 | Phosphotransferase system (PTS) | - | 3 | - | - |
| pac00260 | Glycine, serine and threonine metabolism | - | 3 | - | - |
| pac03030 | DNA replication | - | 3 | - | - |
| pac03410 | Base excision repair | - | 3 | - | - |
| pac00500 | Starch and sucrose metabolism | - | 3 | - | - |
| pac02030 | Bacterial chemotaxis | - | 2 | - | - |
| pac00030 | Pentose phosphate pathway | - | 2 | - | - |
| pac00340 | Histidine metabolism | - | 2 | - | - |
| pac00521 | Streptomycin biosynthesis | - | - | - | 2 |
| pac00680 | Methane metabolism | - | 2 | - | - |
| pac00511 | Other glycan degradation | - | - | - | 2 |
| pac03430 | Mismatch repair | - | 2 | - | - |
| pac03420 | Nucleotide excision repair | - | 2 | - | - |
| pac03020 | RNA polymerase | - | - | - | 2 |
| pac00561 | Glycerolipid metabolism | - | 2 | - | - |
| pac00061 | Fatty acid biosynthesis | - | - | 2 | - |
| pac00471 | D-Glutamine and D-glutamate metabolism | - | - | - | 2 |

*the overview maps pac01100 (metabolic pathways), pac01110 (biosynthesis of secondary metabolites) and pac01120 (microbial metabolism in diverse environments) were omitted.

**Table S3B: Biological processes associated to genes up-regulated in *P. acnes* KPA grown to exponential or stationary growth phase.**

All genes with a fold-change of >2 or <-2 were considered in this analysis. 137 genes were exponential phase (EP) genes, 78 of which (57%) were KEGG-mappable. 122 genes were stationary phase (SP) genes; 38 of which (31%) were KEGG-mappable. Only processes with at least 2 hits are listed.

| **KEGG map** | **Biological process** | **no. EP genes** | **no. SP genes** |
| --- | --- | --- | --- |
| pac01100 | Metabolic pathways | 39 | 22 |
| pac03010 | Ribosome | 24 | - |
| pac00190 | Oxidative phosphorylation | 17 | 2 |
| pac01110 | Biosynthesis of secondary metabolites | 15 | 8 |
| pac01120 | Microbial metabolism in diverse environments | 14 | 7 |
| pac02010 | ABC transporters | 2 | 8 |
| pac00230 | Purine metabolism | 8 | - |
| pac00250 | Alanine, aspartate and glutamate metabolism | - | 7 |
| pac00240 | Pyrimidine metabolism | 4 | 5 |
| pac00330 | Arginine and proline metabolism | 3 | 5 |
| pac00010 | Glycolysis / Gluconeogenesis | 5 | - |
| pac00910 | Nitrogen metabolism | 4 | 4 |
| pac03060 | Protein export | 3 | - |
| pac00680 | Methane metabolism | 3 | 3 |
| pac02020 | Two-component system | 3 | 2 |
| pac03070 | Bacterial secretion system | 3 | - |
| pac00520 | Amino sugar and nucleotide sugar metabolism | 3 | - |
| pac00020 | Citrate cycle (TCA cycle) | 3 | - |
| pac00650 | Butanoate metabolism | 3 | - |
| pac00030 | Pentose phosphate pathway | 3 | - |
| pac03018 | RNA degradation | 3 | - |
| pac00052 | Galactose metabolism | 2 | - |
| pac03030 | DNA replication | 2 | - |
| pac03020 | RNA polymerase | 2 | - |
| pac00051 | Fructose and mannose metabolism | 2 | - |
| pac02060 | Phosphotransferase system (PTS) | - | 2 |
| pac00562 | Inositol phosphate metabolism | - | 2 |
| pac00380 | Tryptophan metabolism | - | 2 |
| pac00900 | Terpenoid backbone biosynthesis | - | 2 |
